# Predicting surgical outcome and sagittal alignment change in patients with cervical spondylosis and degenerative kyphosis after anterior cervical discectomy and fusion

**DOI:** 10.1038/s41598-023-34029-7

**Published:** 2023-04-25

**Authors:** Shaoqing Li, Bingqing Bai, Qiang Li, Qian Yuan, Xiangping Peng

**Affiliations:** 1 Department of Orthopedic Surgery, The Xingtai General Hospital of North China Medical Health Group, No. 202 Bayi Road, Xingtai, 054000 People’s Republic of China; 2 Department of Science and Education, The Xingtai General Hospital of North China Medical Health Group, Xingtai, 054000 People’s Republic of China

**Keywords:** Diseases of the nervous system, Spine regulation and structure, Quality of life, Risk factors, Neurological disorders

## Abstract

The aim of this study was to forecast the risk factors of poor outcomes and postoperative loss of lordosis or recurrence of kyphosis. In this retrospective study, 101 patients with cervical spondylosis and preoperative kyphosis who underwent anterior cervical discectomy and fusion (ACDF) were enrolled, between June 2015 and June 2019. Patients were grouped according to the recovery rate of Japanese Orthopaedic Association (JOA) score whether more than 50%, and the change of postoperative cervical Cobb angle. There were 22 cases with less than 50% of recovery rate and 35 cases with the worsening of postoperative sagittal alignment (WPSA). Multivariate linear-regression analysis was conducted with the data. Advanced age (p = 0.019), longer duration of symptoms (p = 0.003) and loss of local Cobb angle (LCA) after surgery (p = 0.031) was significantly associated with a poor clinical outcome. A whole kyphosis (p = 0.009), aggravated neck pain after surgery (p = 0.012), preoperative lower thoracic 1 (T1) (p** < **0.001), bigger change of C2-7 sagittal vertical axis (SVA) (p = 0.008) and adjacent segment degeneration (ASD) (p = 0.024) was significantly associated with the WPSA. Preoperative health education, nutritional support and early postoperative rehabilitation intervention, in perioperative period, were recommended for patients with advance age, longer duration of symptoms, whole cervical kyphosis and lower T1. Postoperative sagittal malalignment was related to neck pain and ASD after surgery.

## Introduction

Accompanied by the physiologic ageing process, the change of cervical sagittal alignment is a common result, which owes to degenerative changes in the discs and facet joints^1,2^. In addition, people’s professional and personal ways such as long hours of work or study with computers and mobile phones would lead to cervical sagittal malalignment. When a patient develops cervical kyphosis, the decreased cross-sectional area of the spinal canal, distortion of the spinal cord and neck pain may follow^3,4^. Although conservative treatment can alleviate progression of cervical kyphosis, surgery still play an important role in the correction of kyphosis^5,6^. However, it is also controversial whether the correction of kyphosis by an operation can improve the nerves and spinal cord to obtain a good postoperative outcome^7–9^. In addition, in previous studies of cervical anterior surgery, the postoperative less of lordosis and recurrence of kyphosis was rarely mentioned as a complication. Therefore, the purposes of this study were to investigate the predictors of poor outcome, while further to evaluate the risk factors of the worsening of postoperative sagittal alignment (WPSA).

Anterior and posterior decompression operations have been widely performed for patients with cervical spondylosis, which are effective procedures to improve the neurological outcome. However, the optimal surgical strategy in patients with cervical spondylosis particularly associated with kyphosis is debatable. Anterior procedure could adequately and directly remove isolated anterior pathologies, and posterior procedure was required for the treatment of the pathology over many vertebral segments^10,11^. Patients with sagittal kyphotic alignment might appear a poor outcome after posterior laminoplasty, due to insufficient posterior drift of the spinal cord and anterior impingement from kyphotic levels^12,13^. Hence, this investigation reviewed some cases that underwent an anterior cervical discectomy and fusion (ACDF). The object was to predict correlative factors of postoperative outcome and sagittal alignment, focusing especially on change of cervical curvature after surgery.

## Material and methods

### Study design

All procedures were approved by the Ethics Committee of the orthopedics hospital of XingTai, and were carried out in accordance with the relevant guidelines and regulations/ethical principles of the Declaration of Helsinki. We confirmed that informed consent was obtained from all participants. In addition, each patient was informed the risk factors and complications associated with treatment, treatment alternatives before surgery, and signed the written informed consents.

This retrospective study was based on medical records of patients who suffered from cervical spondylosis coexists with preoperative kyphosis. A total of 101 cases (47 males and 54 females) participated, all of whom underwent ACDF between June 2015 and June 2019. Inclusion criteria: patients with cervical kyphosis accompanied by cervical spondylotic radiculopathy (CSR) or cervical spondylotic myelopathy (CSM). The main reasons of cervical spondylosis included ossification of posterior longitudinal ligament, disc herniation and kyphotic alignment. Patients who underwent previous cervical surgery, combined posterior surgery, compression of spina cord or nerves resulting from tumors, fracture, infection, or congenital cervical deformity, missing imaging data and less than 12 months follow up were excluded.

### Radiologic assessment

**Figure 1:**
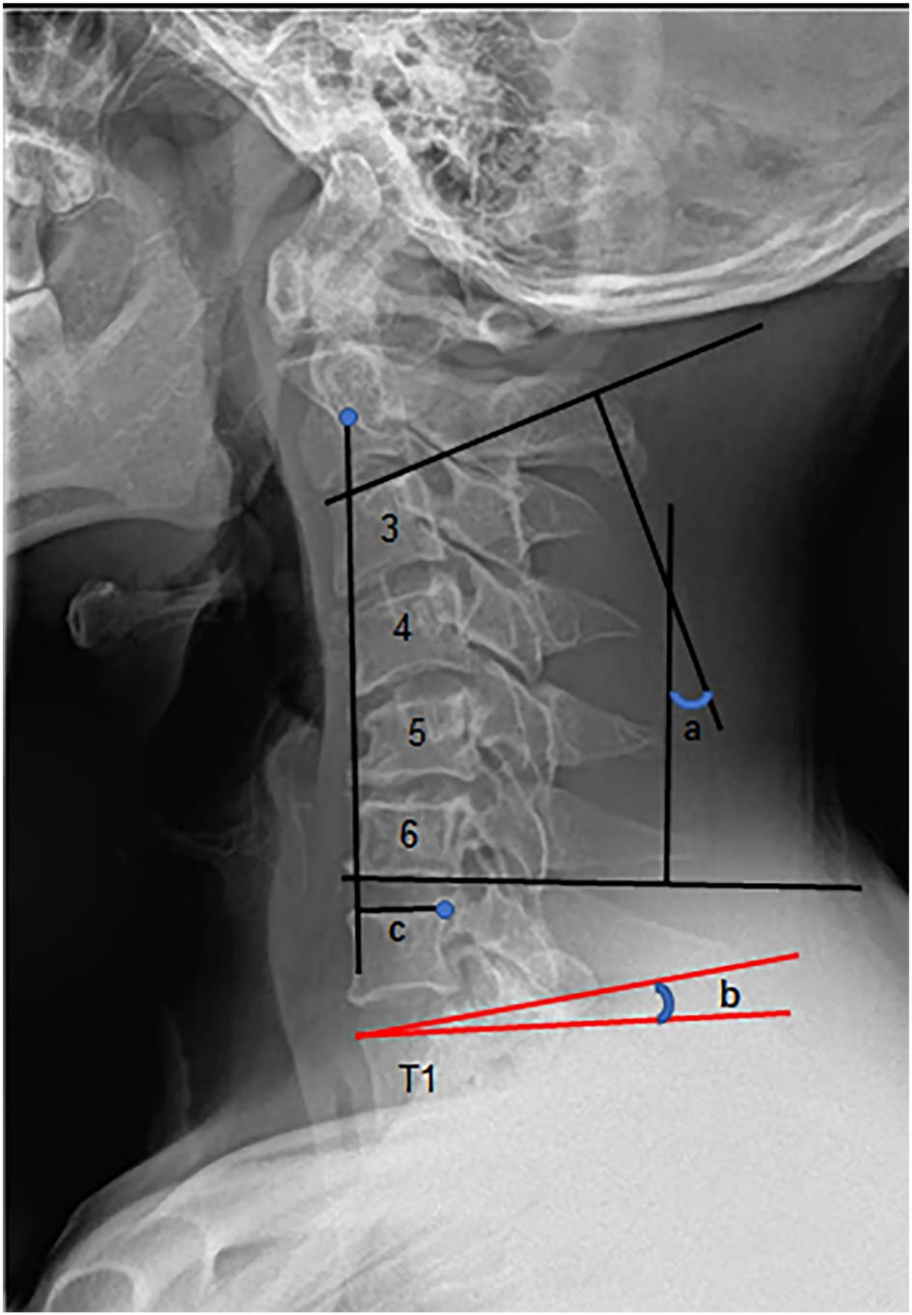
Radiographic measurements: (**a**) local Cobb angle (C3–6), (**b**) T1 slope, (**c**) C2–7 sagittal vertical axis.

The standing lateral radiographs, computed tomography (CT) and magnetic resonance imaging (MRI) were performed for data collection at different stages of treatment. The lordosis and kyphosis were denoted by positive value and negative value, respectively. The C2–7 Cobb angle was defined as the angle of two lines of parallel to the inferior end plates of C2 and C7. Similarly, the local Cobb angle (LCA) was measured between the upper and lower vertebra of kyphotic segment. Hence, the cervical kyphosis was classified as whole type and local type. In addition, Park et al.^14^ reported that the T1 slope was correlated with the cervical sagittal alignment. The T1 slope was defined as the angle between the superior endplate of T1 and a horizontal line. The forward tilt and backward tilt of the superior endplate of T1 were denoted positive value and negative value, respectively. The C2–C7 SVA was defined as the distance between the plumb line of C2 centroid and posterior superior corner of C7^15^. (Fig. 1) In this study, the loss of intervertebral height was measured on CT scan, and the change value more than 3 mm between pre-operation and last follow-up was defined as a noteworthy subsidence^16,17^. The above measurement methods were plotted on the figure. After surgery, less of lordotic alignment or recurrence of kyphosis was defined as the WPSA. The recurrence of kyphosis was defined as the absolute value of the C2–7 Cobb angle or LCA should be greater than 5° at the last follow-up. The diagnostic criteria of adjacent segment degeneration (ASD) included adjacent intervertebral height decreased by more than 3 mm, lower adjacent disc signal on T2-weighted MRI, vertebral posterior spur formation, more than 3 mm newly developed instability. To reduce the measurement errors of image data, the average value of three measurements was implemented. And all data was measured by one rater who was the first author: S.Q.L.

### Clinical assessment

Each patient had a follow-up more than 12 months and was collected data at different times. The JOA score and Visual Analogue Score (VAS) were applied to evaluate the clinical outcome and neck pain degree. The (last follow-up JOA score -preoperative JOA score)/(17 − preoperative JOA score) × 100% was defined as the formula of evaluate clinal outcome. A score ≥ 75% was defined as excellent, ≥ 50% but < 75% as good, ≥ 25% but < 50% as fair, and < 25% as poor. In this study, the patients with recovery rates < 50% would be divided into the poor group. To avoid the interference of postoperative neck incision and muscle injury, the neck pain VAS score collected at 4–6 weeks after surgery.

### Surgical procedure

**Figure 2:**
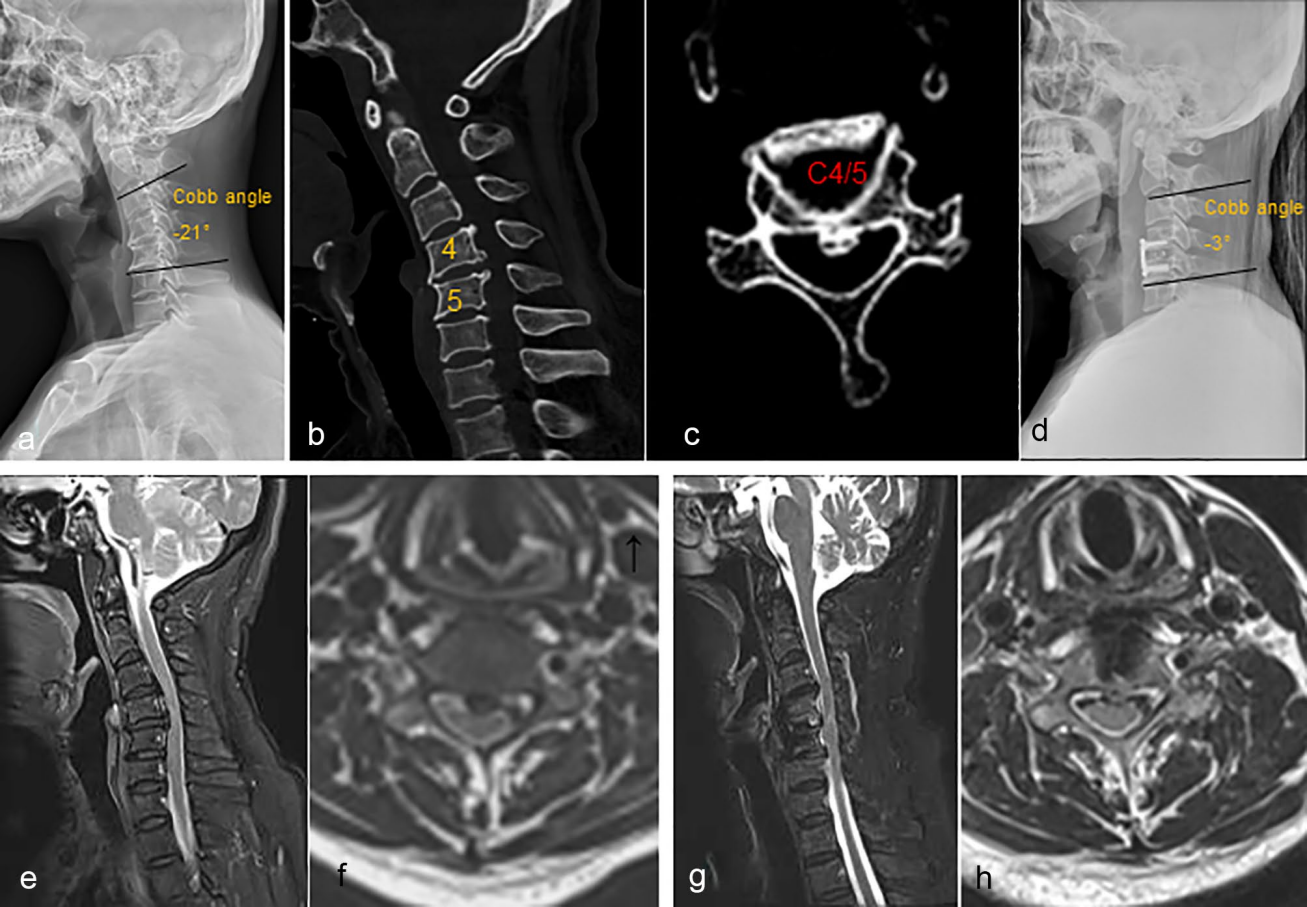
Case 25: a patients was 58 years, male, the walking unstable was main clinical symptom, the compression spinal cord due to herniation of intervertebral disc and cervical kyphosis, X-ray (**a**), CT (**b,c**) and MRI (**e,f**) before the ACDF. We performed single-level ACDF at C4/5, that decompressed spinal cord and corrected the cervical kyphosis (**d,g,h**). The walking unstable gradually improved during postoperative follow-up.

In present study, the operative level was determined by patient’s physical condition, radiography, CT scan, MRI, and neurological examination. The main objective of the ACDF procedure was decompression of the spinal cord or nerves and corrected suitably the kyphosis of the cervical spine. We incised the skin on the right side of the neck and separated the muscle tissue by blunt dissection. After careful hemostasis, reaching the front of responsible vertebra through medial of the carotid sheath. We adequately resected diseased disc and released the uncovertebral joint on both sides. The kyphosis of cervical spinal was corrected by enlarging intervertebral space and changing intraoperative cervical curvature. After the decompression of spinal cord or nerves, an optimal polyether-ether-ketone (PEEK) cage with autogenous bone and anterior fixation of plate system were implant in an appropriate location. Finally, we sutured the incision and observed closely the oozing of blood and breathing state within 48 h after surgery (Fig. 2).

### Statistical analyses

The means and standard deviations and frequencies were used to describe continuous variables and categorical variables, respectively. And the independent variables were analyzed by Mann–Whitney U-test, independent t-test and Chi-squared test. Multiple linear regression was applied to predict the correlation between the independent variables with a p-value less than 0.05 and dependent variable. The p < 0.05 represents a statistically significant difference. All analyses were performed using version SPSS software (version 22.0; SPSS Inc., Chicago, IL).

## Results

A total of 101 cases were reviewed in this study, including 58 CSR patients and 43 CSM patients. There were 13 cases, 57 cases and 31 cases who underwent one segment, two segments and three segments ACDF procedure, respectively. By comparison of JOA score between pre-operation and last-follow, 22 cases were divided to the poor group with the recovery rates of JOA score greater less than 50%. Compared with the preoperative JOA score (10.96 ± 2.33), the JOA score at last follow-up (14.18 ± 1.95) had a statistically significant improvement (p < 0.001). There was no difference between the groups in terms of preoperative JOA score (p = 0.449), T1 slope (p = 0.189), and postoperative implant subsidence (p = 0.818) and ASD (p = 0.190). However, age (p = 0.002), duration of symptoms (p < 0.001), Disease classification (p = 0.024) and change of LCA after surgery (p = 0.011) was significantly difference between two groups (Table 1).

**Table 1:** Comparison of patient characteristics between good group and poor group.

	Good (n = 79)	Poor (n = 22)	*p*
Demographics			
Age (years)	55.70 ± 5.42	60.18 ± 5.58	**0.002**
Female, n	41	13	0.550
BMI (kg m^−2^)	22.86 ± 3.9	24.33 ± 4.21	0.529
Duration of symptoms (m)	8.67 ± 7.53	10.33 ± 9.82	** < 0.001**
Disease classification (CSR: CSM), n	50:29	8:14	**0.024**
Surgical level (1:2:3), n	8:47:24	5:10:7	0.255
Classification of kyphotic alignment			0.596
Whole, (n)	30	7	
Local, (n)	49	15	
JOA score			
Preoperative	11.04 ± 2.0	10.68 ± 2.38	0.449
Postoperative (4–6 weeks)	14.72 ± 1.06	12.32 ± 1.86	** < 0.001**
Last follow-up	14.83 ± 1.06	12.55 ± 1.65	** < 0.001**
Recovery rate (pre- and last), %	63.82 ± 12.22	27.41 ± 13.37	** < 0.001**
Radiographic parameters			
Pre-T1 slope (°)	14.14 ± 7.11	15.86 ± 8.54	0.189
C2–7 SVA (mm)			
Preoperative	17.87 ± 9.30	19.45 ± 8.06	0.499
Postoperative	15.26 ± 10.19	16.18 ± 12.31	0.725
Last follow-up	14.72 ± 11.27	17.11 ± 8.29	0.145
Change value (post- and last)	2.36 ± 1.68	1.93 ± 2.81	0.278
C2–7 Cobb angle (°)			
Preoperative	−9.27 ± 11.72	−7.68 ± 10.78	0.371
Postoperative	5.02 ± 9.42	5.43 ± 8.70	0.694
Last follow-up	3.87 ± 10.68	5.91 ± 8.83	0.218
Change value (post- and last)	−1.62 ± 1.77	0.66 ± 2.53	0.198
LCA (°)			
Preoperative	−15.73 ± 4.42	−17.31 ± 5.02	0.096
Postoperative	7.89 ± 9.23	9.22 ± 7.38	0.552
Last follow-up	6.31 ± 7.72	5.66 ± 6.35	0.103
Change value (post- and last)	−1.59 ± 1.45	−2.87 ± 2.63	**0.011**
Complication, (n)			
Implant subsidence > 3 mm	6	2	0.818
ASD	12	5	0.190

*BMI* body mass index, *CSR* cervical spondylotic radiculopathy, *CSM* cervical spondylotic myelopathy, *JOA* Japanese Orthopaedic Association, *T1* thoracic 1, *SVA* sagittal vertical axis, *LCA* local Cobb angle, *ASD* adjacent segment degeneration.

Significant values are in bold.

The multiple linear regression analyses predicted the risk factors of poor outcome, which showed a significant regression (p = 0.001) with an R^2^ of 0.168 and an adjusted R^2^ of 0.157. The model revealed that advance age (p = 0.019), longer duration of symptoms (p = 0.003) and loss of LCA after surgery (p = 0.031) was significant predictors of poor outcome (Table 2).

**Table 2:** Multiple linear regression model for predictive factors of recovery rate JOA score after ACDF.

	*B*	*S.E. Beta*	*Beta*	*t*	*p*
Age (years)	−0.811	0.370	−0.236	−2.393	**0.019**
Duration of symptoms (m)	−0.363	0.126	−0.431	−3.526	**0.003**
Disease classification (0 = CSR, 1 = CSM)	−5.315	3.917	−0.136	−1.375	0.172
Change value of LCA (post- and last)	−1.294	0.902	−0.172	−1.926	**0.031**

*CSR* cervical spondylotic radiculopathy, *CSM* cervical spondylotic myelopathy, *LCA* local Cobb angle.

Significant values are in bold.

The less of lordosis and even recurrence of kyphosis occurred in some patients after surgery. Based on the change of sagittal alignment, the patients were divided to the maintained group and worsening group (Table 3). There was significant difference between the groups in terms of age (p = 0.031), classification of kyphosis (p = 0.002), recover rate of JOA score (p = 0.016), neck pain VAS score at last follow-up (p = 0.003), the change of neck pain VAS score (p = 0.007), preoperative T1 slope (p < 0.001), C2–7 SVA at last follow-up (p = 0.005), the change of C2–7 SVA after surgery (p = 0.001), and postoperative implant subsidence (p = 0.001) and ASD (p = 0.005). In addition, to determine the relationship between ASD and neck pain, change of neck pain VAS score between 4 and 6 weeks after surgery and last-follow was compared, which was 1.63 ± 1.21 and 1.46 ± 1.01 in patients with ASD or not, respectively (p = 0.183).

**Table 3:** Comparison of patient characteristics for the change of sagittal alignment after operation.

	Maintained (n = 66)	Worsening (n = 35)	***p***
Demographics			
Age (years)	56.32 ± 5.87	59.21 ± 5.68	**0.031**
Female, n	35	19	0.904
BMI (kg m^−2^)	25.16 ± 4.69	23.72 ± 4.61	0.429
Duration of symptoms (m)	8.13 ± 8.53	9.33 ± 8.01	0.661
Disease classification (CSR: CSM)	37:29	21:14	0.703
Surgical level (1:2:3)	10:39:17	3:18:14	0.607
Classification of kyphotic alignment			**0.002**
Whole, (n)	17	20	
Local, (n)	49	15	
JOA score			
Preoperative	10.17 ± 3.08	11.64 ± 1.72	0.646
Postoperative (4–6 weeks)	12.95 ± 2.26	13.61 ± 1.46	0.811
Last follow-up	13.05 ± 1.83	13.83 ± 2.15	0.283
Recovery rate (pre- and last), %	57.82 ± 17.91	54.15 ± 16.47	**0.016**
Neck pain VAS score			
Preoperative	4.96 ± 2.41	5.33 ± 3.02	0.472
Postoperative (4–6 weeks)	1.93 ± 0.97	2.04 ± 1.23	0.751
Last follow-up	2.23 ± 1.17	3.11 ± 1.48	**0.003**
Change value (post- and last)	1.55 ± 0.56	1.72 ± 1.07	**0.007**
Radiographic parameters			
Pre-T1 slope (°)	17.82 ± 9.53	13.14 ± 7.25	** < 0.001**
C2–7 SVA (mm)			
Preoperative	18.11 ± 11.88	20.27 ± 10.27	0.239
Postoperative	14.33 ± 9.19	15.09 ± 11.71	0.472
Last follow-up	15.88 ± 8.24	18.81 ± 10.29	**0.005**
Change value (post- and last)	1.44 ± 1.46	3.51 ± 2.36	**0.001**
C2–7 Cobb angle (°)			
Preoperative	−8.33 ± 10.82	−8.92 ± 11.78	0.771
Postoperative	5.27 ± 6.51	6.32 ± 5.42	0.694
Last follow-up	5.83 ± 7.99	4.47 ± 8.51	0.222
Change value (post- and last)	1.19 ± 2.02	−2.51 ± 3.17	**0.001**
LCA (°)			
Preoperative	−15.36 ± 7.08	−16.31 ± 6.12	0.373
Postoperative	8.02 ± 8.42	9.51 ± 6.74	0.241
Last follow-up	10.37 ± 5.43	5.86 ± 6.22	**0.018**
Change value (post- and last)	3.27 ± 2.52	−4.73 ± 3.56	** < 0.001**
Complication, (n)			
Implant subsidence > 3 mm	1	7	**0.001**
ASD	6	11	**0.005**

*BMI* body mass index, *CSR* cervical spondylotic radiculopathy, *CSM* cervical spondylotic myelopathy, *JOA* Japanese Orthopaedic Association, *VAS* Visual Analogue Score, *T1* thoracic 1, *SVA* sagittal vertical axis, *LCA* local Cobb angle, *ASD* adjacent segment degeneration.

Significant values are in bold.

The multiple linear regression analyses predicted the risk factors of the WPSA, which showed a significant regression (p < 0.001) with an R^2^ of 0.302 and an adjusted R^2^ of 0.266. The model showed that patient with the whole kyphosis (p = 0.009), preoperative lower T1 slope (p** < **0.001), aggravated neck pain (p = 0.012) and bigger C2–7 SVA (p = 0.008) after surgery, and ASD (p = 0.024) was significant predictors of the WPSA (Table 4).

**Table 4:** Multiple linear regression model for predictive factors of worsening sagittal alignment after operation.

	*B*	*S.E. Beta*	*Beta*	*t*	*p*
Age (years)	−0.121	0.079	−0.140	−1.531	0.129
Recovery rate of JOA score (pre- and last)	0.041	0.025	0.157	1.686	0.095
Classification of kyphotic alignment (0 = whole, 1 = local)	2.619	0.982	0.245	2.666	**0.009**
Neck pain VAS score					
Last follow-up (1 year)	−0.723	0.379	−0.178	−1.907	0.060
Change value (pre- and last)	−0.511	0.348	−0.221	−2.327	**0.012**
Pre-T1 slope (°)	0.223	0.061	0.341	3.689	** < 0.001**
C2–7 SVA (mm)					
Last follow-up	−0.081	0.045	−0.153	−1.793	0.076
Change value (post- and last)	−0.102	0.061	−0.252	−2.725	**0.008**
Implant subsidence > 3 mm (0 = occurred, 1 = on occurred)	2.189	1.767	0.122	1.239	0.218
ASD (0 = occurred, 1 = on occurred)	2.315	1.010	0.216	2.292	**0.024**

*JOA* Japanese Orthopaedic Association, *VAS* Visual Analogue Score, *T1* thoracic 1, *SVA* sagittal vertical axis, *ASD* Adjacent segment degeneration.

Significant values are in bold.

## Discussion

This part focuses on the risk factors of poor outcome, the relationship of T1 slope and the WPSA, influence of implant subsidence, the relationship of ASD and cervical kyphosis and the relationship of chronic pain and the WPSA. An optimal surgical approach and adequate decompression of spinal cord and nerves are the precondition of good postoperative outcome. The maintain of cervical lordosis have be considered an important factor for the long-term clinical outcome^7^. Moreover, Kim et al.^3^ showed that the less of lordosis after surgery would contribute to the reduction of relative cross-sectional area of spinal canal. However, Kaptain et al.^8^ deem clinical outcome was not association with the postoperative sagittal alignment. It remained controversial that the relationship between the correction of kyphosis and the improvement of neurological functions. In present study, the change of postoperative LCA was significantly related to the poor outcome, but the change of postoperative C2–7 Cobb was not associate with the poor outcome. Because of the effect of sigmoid alignment on the C2–7 Cobb, we thought that the LCA was more suitable for evaluation of the spinal cord compression. In addition, age and duration of symptoms were considered as predictors of poor outcome after surgery.

The T1 slope was supposed to be an important information of cervical curvature correction^14^. In previous study, T1 slope was frequently used as a risk factor to predict the less of lordosis after laminoplasty^18,19^. However, it was not usually evaluated in patient who underwent ACDF. In this research, the WPSA was significantly related to preoperative lower T1 slope, especially when the T1 slope was negative value before surgery. There were 17 patients with a negative value, and 14 of them happened the WPSA. By comparing the change of T1 slope angle before and after surgery, the T1 slope angle was relatively stable in this study. According to this conclusion, we considered that ACDF operation could correct the local kyphosis, but to ensure sagittal balance of the cervical spine, that a low T1 slope angle required a small lordosis of the lower cervical spine. Hence, the preoperative lower T1 slope might contributed to the WPSA.

There were eight patients occurred the implant subsidence in this study. Lee et al.^20^ thought that the lower T1 slope was a risk factor of implant subsidence. Suh et al.^21^ thought that cage morphology and material composition were related to the implant subsidence. However, we more likely to consider the early fusion was the determinant of implant subsidence. The results showed that the implant subsidence was not significantly related to the WPSA by multiple linear regression. The relatively small number of patients with cage subsidence might affect the result. In addition, there was a significant correlation between the whole type of cervical kyphosis and the WPSA. This reason might be a certain degree of limitation of anterior surgery on overall correction of the cervical kyphosis.

The intervertebral space narrowing was one of the important causes of postoperative ASD, while was an inevitable result of implant subsidence. In previous studies, Barsa et al.^22^ thought implant subsidence could accelerate cervical degenerative changes in adjacent segments. Lazic et al.^23^ showed that intervertebral spaces were positively correlated with cervical lordosis. Hence, we searched the relationship between the ASD and change of postoperative cervical curvature. The present result showed that the patients with ASD would contribute to the less of lordosis and even recurrence of kyphosis after surgery. This was related to the cause of ASD formation, such as instability and stress change in the adjacent motion segments. In addition, Risbud et al.^24^ found that degeneration of the intervertebral discs was a major contributor to neck pain, which physiologically based. However, the occurrence of ASD after surgery did not provide a powerful evidence for chronic neck pain in this study.

Chronic neck pain is a common symptom in patient with cervical vertebra degeneration^24^. Relieving neck pain could improve patient’s the quality of life and postoperative satisfaction. However, it remained controversial whether there was a connection between the neck pain and sagittal malalignment. Grob et al.^25^ thought cervical structural abnormalities was not necessarily indicative of the cause of pain. Whereas, Ferch et al.^4^ thought that neck pain could be alleviated by correcting and maintaining the physiological lordosis of cervical spine. In addition, some scholars found that there was a significant relationship between cervical muscular imbalance and the loss of physiological lordosis^26^. And the muscular imbalance was the one of important factors of choric pain. Based on the viewpoints of previous studies, we further investigated whether postoperative chronic pain was related to the WPSA. The results showed that the less of lordosis or recurrence of kyphosis after surgery would contribute to persistent neck pain.

In this study, several limitations should be presented. Firstly, this was a single-center retrospective study, and 12 months of follow-up was relatively short. However, a longer follow-up did not obtain a significant change of clinical and radiologic over 24 months after 6 months^27^. Secondly, the SF-36 scale and the HR-QOL scale were not applied to the evaluation of postoperative outcome. Thirdly, this study was not considered the correlation between the cervical sagittal alignment and thoracolumbar or spino-pelvic parameters. Fourthly, it is worthful to the orientation of study by investigating the relationship between postoperative outcome and clinical supports which including perioperative nutritional support and anti-osteoporosis therapy. Finally, all the imaging data were measured by one rater, and the intra-rater errors were inevitable. Although several limitations were included in this study, preoperative T1 slope and type of cervical kyphosis were meaningful preoperative parameters for predicting the change of postoperative cervical curvature. The results could helpful to improve the postoperative outcome and to determine whether anterior procedure was optimum selection.

## Conclusion

Preoperative health education, nutritional support and early postoperative rehabilitation intervention, in perioperative period, were recommend for patients with advance age, longer duration of symptoms, whole cervical kyphosis and lower T1. In addition, postoperative sagittal malalignment was related to neck pain and ASD after surgery.

## Data Availability

The datasets used and/or analysed during the current study available from the corresponding author on reasonable request.
